# Autoimmune Encephalitis in Acute Care—Pathology, Diagnosis, and Management

**DOI:** 10.1002/advs.202519049

**Published:** 2026-03-29

**Authors:** Suneesh Thilak, David Okoh, William Scotton, Shanika Samarasekera, Vikram Patil, Suresh Renukappa, Andrew Macduff, Subashini Suresh, Rajeev Krishnadas, Tonny Veenith

**Affiliations:** ^1^ Department of Anaesthesia Critical Care and Acute Care Royal Wolverhampton NHS Trust Wolverhampton United Kingdom; ^2^ Department of Neurology Royal Wolverhampton NHS Trust Wolverhampton United Kingdom; ^3^ Department of Neurology University Hospitals Birmingham NHS Foundation Trust Birmingham United Kingdom; ^4^ Department of Radiology JSS Medical College JSS AHER Mysuru India; ^5^ JSS AHER – Wolverhampton Centre For Future Health and Policy Innovation Mysuru India; ^6^ Faculty of Science and Engineering University of Wolverhampton Wolverhampton United Kingdom; ^7^ Department of Psychiatry University of Cambridge Cambridge United Kingdom; ^8^ Research Institute of Healthcare Sciences (RIHS) University of Wolverhampton Wolverhampton United Kingdom

**Keywords:** autoimmune encephalitis, critical care, neuroimmunology, status epilepticus, immunotherapy, neurological emergencies, prognosis, neurocritical care

## Abstract

Autoimmune encephalitis (AE) is characterized by immune‐mediated inflammation of the brain parenchyma, presenting with various neurological syndromes, including but not limited to seizures, altered consciousness, neuropsychiatric symptoms, and movement disorders. A significant proportion of patients with AE develop life‐threatening complications that require hospital and potentially ICU admission. These patients present unique diagnostic and therapeutic challenges, presenting with a range of neurological emergencies such as refractory status epilepticus, severe dysautonomia, coma, and respiratory failure. Diagnosis relies on a combination of clinical criteria, detection of autoantibodies in serum and cerebrospinal fluid, neuroimaging, and electroencephalography, though antibody‐negative AE poses considerable diagnostic difficulty. Management is centered on prompt initiation of first‐line immunotherapies (corticosteroids, plasma exchange, and intravenous immunoglobulin therapy) and escalation to second‐line or emerging targeted therapies (e.g., rituximab, cyclophosphamide, IL‐6 inhibitors, proteasome inhibitors) in refractory cases, alongside aggressive supportive care for neurological and systemic complications. The prognosis is variable and influenced by factors such as the specific autoantibody, the timeliness of treatment, and the severity of complications. Long‐term sequelae, including cognitive and psychiatric impairments, are common among survivors. This review provides a synthesis of current knowledge on AE in acute and intensive care. Given these diagnostic challenges and the potential for severe symptom presentation, the authors believe that this review is an essential addition to the discourse. This review covers pathophysiology, epidemiology, clinical manifestations, diagnostic approaches, immunotherapeutic strategies, management of critical complications, syndrome‐specific considerations, prognostication, intensive care unit‐related complications, and future research priorities to support optimal care.

## Graphical abstract

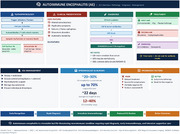

## 1 Introduction

Autoimmune encephalitis (AE) is an expanding category of immune‐mediated diseases affecting the central and the peripheral nervous system (CNS). AE presentations range rom cognitive decline to severe, refractory encephalopathy [1]. Over the past two decades, AE has gained prominence as a significant cause of noninfectious encephalitis, a shift largely driven by the discovery of an increasing number of specific neuronal autoantibodies and a growing awareness among clinicians [2]. Historically, a substantial number of these cases were likely categorized as “encephalitis of unknown origin,” accounting for 30%–48% of all cases [3]. This understanding suggests a shift in which AE is no longer viewed as a collection of rare disorders but as a diagnostic consideration in patients presenting with acute or subacute neurological dysfunction with unclear etiology. This requires a consideration of AE in unexplained acute neurological deterioration, particularly within acute settings.

A substantial number of patients develop severe neurological complications that necessitate admission to an intensive care unit (ICU) [4]. Estimates suggest that 30%–40% of individuals with antibody‐negative AE and between 55% and 70% of patients in broader AE cohorts require ICU‐level care during their illness [5, 6]. Such admissions are frequently associated with increased morbidity, prolonged hospital stays, and higher mortality rates, often due to poor overall prognosis [5, 6, 7]. The high rate of ICU admission and the associated adverse outcomes highlight a critical unmet need for more rapid diagnostic modalities and highly effective, early‐stage interventions that are validated explicitly in the critically ill AE population. It is essential to identify strategies to prevent such admissions and shorten their duration by accelerating diagnosis and enhancing treatment efficacy.

Critically ill patients with AE pose diagnostic and therapeutic challenges. Clinical presentations may be nonspecific, confounded by sedation or coexisting critical illnesses and overlap with other conditions such as sepsis, metabolic or infective encephalopathies [2]. Management requires prompt initiation of immunotherapy, aggressive supportive care for life‐threatening neurological emergencies (e.g., status epilepticus, autonomic storm), and surveillance to mitigate the complications arising from both the disease and its treatment [4]. In addition, the inherent heterogeneity of AE syndromes is driven by different antibody targets and pathophysiological mechanisms [1]. This results in considerable variation in treatment pathways, rather than a syndrome‐specific management approach.

## 2 Aims

This review aims to provide a comprehensive overview of AE in the ICU. It will explore the pathophysiology and diagnostic strategies underpinning the clinical spectrum of AE leading to critical illness. We will focus on immunotherapeutic interventions, ranging from first‐line agents to emerging therapies inrefractory patients, as well as the management of their associated complications. The review will also cover the management of specific neurological emergencies, considerations for distinct AE syndromes frequently encountered in the ICU, prognostication, and the trajectory of recovery and long‐term management following ICU discharge. Finally, it will highlight recent advancements, ongoing research, and future directions to address the current knowledge gaps and improve outcomes for this vulnerable patient population.

### 2.1 Pathophysiological Hallmarks and Classification of AE

**FIGURE 1:**
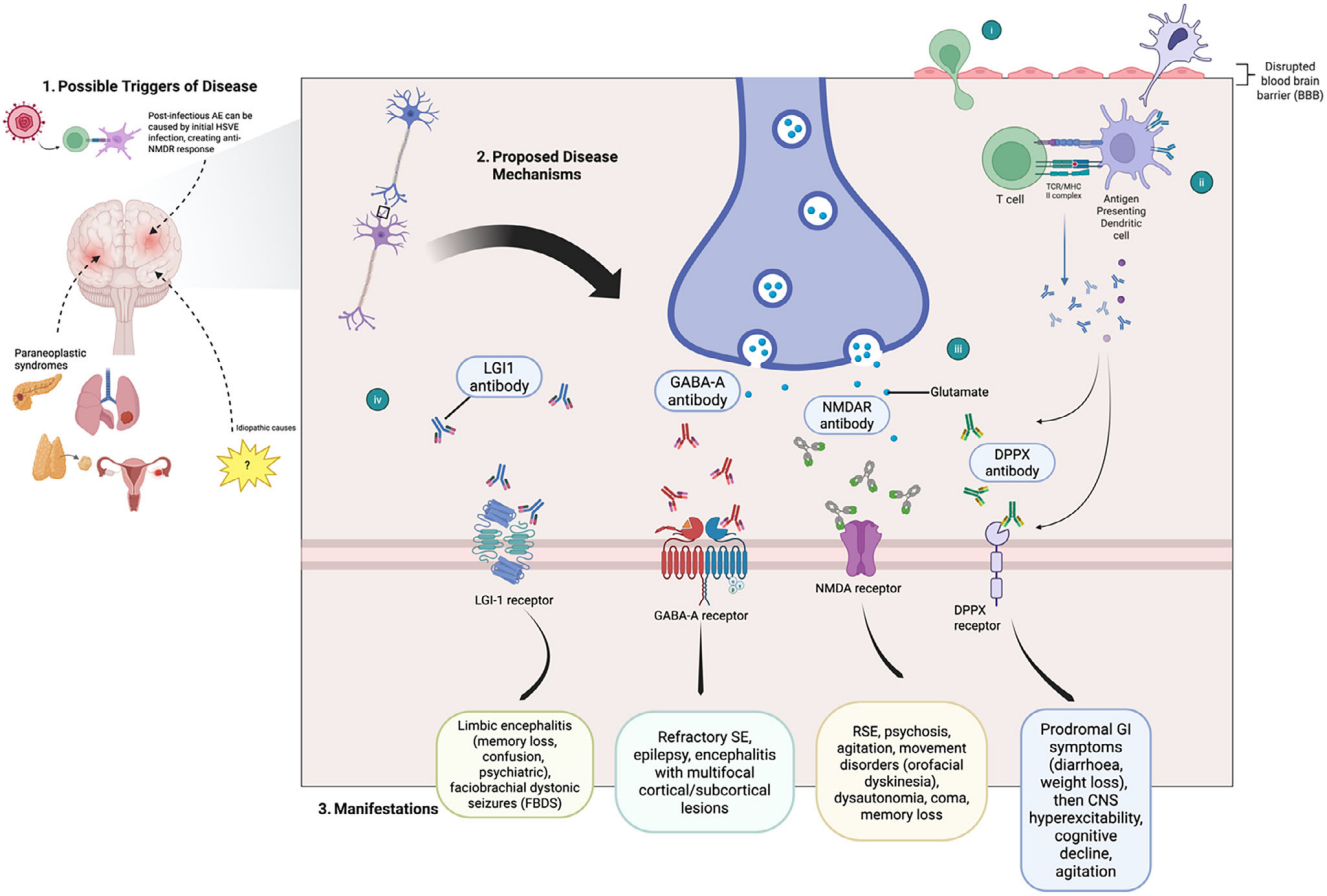
Proposed pathogenesis of AE, including triggers and examples of antibody‐positive disease manifestations. Other key antibodies included in the screen are found in the table below (e.g., Caspr2, GAD65, GABA_B_, AMPAR, and others). Overview of the steps of the proposed disease mechanism as described in the above figure: i) The blood‐brain barrier becomes permeable to leukocytes, allowing them to enter into the central nervous system; ii) T cells interact with antigen‐presenting dendritic cells; iii) antibodies and cytokines are released into synaptic junctions; iv) antibodies bind, causing dysfunction of channel receptor proteins. Figure 1 was created with BioRender.com, released under a Creative Commons Attribution‐NonCommercial‐NoDerivs 4.0 International license Veenith, T. (2026) https://BioRender.com/zi34cyt.

AE is characterized by an abnormal immune response targeting neuronal self‐antigens (summerised in Figure 1). The underlying mechanisms involve either direct antibody‐mediated neuronal dysfunction or T‐cell‐mediated neuronal injury, often triggered by infections or underlying neoplasms [8, 9].

Autoantibodies targeting intracellular antigens, also known as onconeural antibodies (e.g., anti‐Hu, anti‐Yo, anti‐Ma2), are typically epiphenomena that serve as markers of a paraneoplastic neurological syndrome [8, 9]. In these patients, the primary mechanism is believed to be a T‐cell‐mediated cytotoxic response to neurons, resulting in irreversible neuronal death [8, 9]. Glutamic acid decarboxylase 65 (GAD65) antibodies are somewhat unique; GAD65 is an intracellular enzyme, but AE associated with these antibodies can sometimes respond to immunotherapy, although often requiring more intensive or prolonged treatment courses [4].

The distinction between cell surface antigen‐mediated and intracellular antigen‐associated AE is critical in the ICU, as it has direct implications for prognosis and the anticipated aggressiveness and efficacy of immunotherapy [8]. While patients with either type may present with severe neurological crisis requiring ICU admission, the expectation of neurological recovery and the specific immunotherapeutic strategies may differ. For instance, the identification of intracellular antigens might shift the focus more rapidly towards comprehensive tumor screening and potentially different immunomodulatory approaches, while acknowledging a higher likelihood of irreversible neuronal damage. Conversely, cell surface antigen‐associated AE requires a potentially prolonged immunotherapy with a greater expectation of functional recovery, even after a severe ICU course [8] (Figure 1).

Triggers for AE are varied. Post‐infectious AE can occur following infections, for example, herpes simplex virus encephalitis (HSVE) has been reported to trigger N‐methyl‐d‐aspartate receptor (NMDAR) antibodies [10], suggesting a “two‐hit” model relevant for ICU diagnostics [2]. In such cases, an initial infectious workup may be positive, but a secondary autoimmune process can emerge, prolonging or worsening the ICU length of stay. This necessitates ongoing monitoring for autoimmune conversion even after an initial infectious diagnosis is established, as continued antimicrobial therapy alone would be insufficient. Several key autoantibody syndromes are frequently encountered in the ICU due to the severity of their manifestations: NMDAR‐antibody encephalitis often presents with a multistage illness that includes psychiatric disturbances, seizures, movement disorders, autonomic instability, and progression to coma [3, 11]. LGI1‐antibody encephalitis typically causes limbic encephalitis [12] with short‐term memory loss, psychiatric features, and seizures, with faciobrachial dystonic seizures (FBDS) and hyponatremia being common [12, 13]. CASPR2‐antibody encephalitis can manifest as limbic encephalitis, Morvan syndrome, and neuromyotonia [4]. Gamma‐aminobutyric acid type A receptor (GABA_A_R)‐antibody encephalitis is characterized by prominent, often refractory, seizures and status epilepticus [11]. Gamma‐aminobutyric acid type A receptor (GABA_B_R)‐antibody encephalitis also presents with severe seizures and limbic encephalitis and has a high association with small‐cell lung cancer (SCLC) [4]. The diversity of these antibody targets and their specific molecular effects implies that even within cell surface antigen‐mediated AE, the precise nature of neurological emergencies observed in the ICU could differ, potentially guiding more antibody‐specific supportive care strategies.

### 2.2 Epidemiology and ICU Burden of Autoimmune Encephalitis

The epidemiological impact of AE, particularly the burden it places on intensive care, is now recognized as substantial, although the precise scope is still under investigation. Estimates suggest an annual incidence of all‐cause encephalitis reaching up to 12.6 per 100,000 individuals, with approximately 20%–30% of these cases believed to have an autoimmune origin [4]. Population‐based studies have indicated a prevalence of AE around 13.7 per 100,000 people, a figure comparable to the combined prevalence of all infectious encephalitides [4]. This figure may even be an underestimation due to historical labelling of cases as idiopathic and ongoing advancements in diagnostic capabilities [4]. AE is a substantial and likely under‐recognized component of neurocritical care. This has significant implications for the allocation of ICU resources. It underscores the vital necessity for easy access to specialist consultations across neurology, rheumatology, and immunology. In addition, it emphasizes the critical need to enhance training for ICU staff, specifically in managing these complex cases [3]. Nearly 70% of patients require intensive care during the index admission, including 30%–40% of antibody‐negative cases, and stays are prolonged (mean ICU length of stay is 22 days) [3, 4, 11]. These demands have implications for resource allocation, which requires access to neurology, rheumatology, and immunology opinions, as well as the delivery of immunotherapy and the prevention of disease‐ and treatment‐related complications (Table 1).

**TABLE 1:** Leading clinical factors resulting in admission to intensive care.

Key Risk Factors That Increase Likelihood of ICU Admission	Clinical Factors That Necessitate ICU Admission
1) Long symptom duration prior to hospitalization [4].2) Severe hyperkinetic movement disorders for patient safety, management of secondary complications, or the need for deep sedation. These can include: Severe dyskinesias Dystonia Catatonia [4] 3) Higher scores on disease severity scales like the Clinical Assessment Scale in Autoimmune Encephalitis (CASE) or the modified Rankin Scale (mRS) at hospital admission [14].4) Other risk factors: Antibody negative AE Anemia [4] Failure of first‐line immunotherapy [14] Definitive diagnosis of AE [12] Cerebrospinal fluid (CSF) white blood cell (WBC) count >20 cells/mm^3^ [13]	i) Severe neurological complicationsIn the Qin et al. (2025) (134 ICU patients) study this accounted for 64.2% [15], whereas in Harutyunyun et al. (2017) (32 ICU patients) it was 54%. These included: Status epilepticus (SE) Refractory status epilepticus ii) Severe autonomic dysfunction, e.g.: Life‐threatening cardiovascular instability Malignant arrhythmias Severe hypertension/hypotension Profound hyperthermia iii) Respiratory failure resulting in need for mechanical ventilation. Accounts for 26.8%* in one AE group [15]. Also accounts for 23.4% in a cohort of antibody‐negative AE patients [3]. Can be caused by: [2] Depressed consciousness Centrally mediated hypoventilation Breathing difficulties due to seizures Bulbar dysfunction iv) Other factors that may require intensive monitoring or airway protection in ICU: [2] Altered mental status Encephalopathy Elevated intracranial pressure [4] Coma

Enhanced early recognition of AE in outpatient or general hospital settings could facilitate timely treatment. Critical illness is seen at a greater rate in patients with delays between the first onset of symptoms and hospital admission [16]. Thus, early recognition can reduce the incidence of critical illness and deterioration requiring ICU admission, thereby mitigating associated morbidity and healthcare costs [16, 17, 18]. The retrospective study carried out by Cohen et al. highlighted the substantive cost of this demographic of patients [17].

**FIGURE 2:**
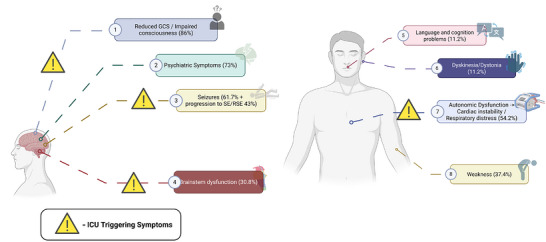
Overview of common symptoms of AE with frequency of presentation as suggested by Li et al. (2025).

A challenging subset of patients is those with antibody‐negative AE, significant proportion require intensive care [3]. Their management heavily relies on clinical suspicion and the exclusion of other diagnoses, which can delay the initiation of specific immunotherapy and complicate prognostic discussions within the ICU. This underscores the urgent need for improved biomarkers that go beyond currently available antibody panels to mitigate delays in hospital admission (Figure 2).

### 2.3 Clinical Spectrum and Neurological Emergencies in Critically Ill AE Patients

Critically ill patients with AE often exhibit a subacute onset of neurological symptoms, evolving over weeks to less than three months. These symptoms may include memory deficits, altered mental status, behavioral changes, psychosis, or seizures [2]. The clinical manifestations in the ICU are diverse and frequently severe (Figure 2), reflecting the specific neuroanatomical location targeted by the immune‐mediated attack [1]. A study of antibody‐negative AE patients in the ICU revealed common findings such as impaired consciousness (86%), psychiatric symptoms (73.8%), seizures (61.7%, with 43% experiencing status epilepticus), dyskinesia or dystonia (11.2%), language problems (11.2%), brainstem dysfunction (30.8%), weakness (37.4%), and autonomic dysfunction (54.2%) [2, 3]. The co‐occurrence of multiple, severe neurological emergencies presents a unique management challenge in the ICU, often signaling a widespread and aggressive immune assault that leads to high morbidity and mortality.

Seizures are a hallmark of many AE syndromes, and their progression to status epilepticus (SE) or refractory status epilepticus (RSE) is a primary reason for ICU admission [4]. AE is increasingly recognized as a frequent cause of new‐onset refractory status epilepticus (NORSE). NORSE is defined as SE in patients without prior history of seizures or a readily identifiable acute or active structural, toxic, or metabolic cause. AE is the commonest cause, accounting for at least 20% of these cases [4, 19]. RSE, defined as SE that persists despite adequate trials of first‐ and second‐line anti‐seizure medications (ASMs), affects a significant proportion of AE patients in the ICU [4]. The management of AE‐associated RSE poses the dual challenge of suppressing seizures using increasingly potent ASMs, potentially including anesthetic‐induced burst suppression, while aggressively treating the underlying immune dysregulation with concurrent immunotherapy [4]. This dual focus is unique to AE‐associated RSE and necessitates close collaboration between intensivists and neurologists/immunologists.

Severe autonomic dysfunction and cardiovascular instability are frequent and potentially life‐threatening complications in ICU‐managed AE patients [20]. These complications can manifest as profound cardiovascular instability, including labile blood pressure (hypertensive crises or severe hypotension), tachyarrhythmias or bradyarrhythmias (sometimes requiring pacemaker insertion), hyperthermia or hypothermia unrelated to infection, central hypoventilation or hyperventilation, gastrointestinal dysmotility (ileus, diarrhea), hypersalivation, and hyperhidrosis [8]. The presence of autonomic instability not only poses a management challenge but also predicts a poor response to first‐line immunotherapy [4] and is associated with worse outcomes [20]. This suggests that dysautonomia is a core manifestation of the disease process, reflecting immune attack on critical autonomic control centers in the brainstem and hypothalamus.

Many AE patients in the ICU experience a significant depression in their level of consciousness, ranging from lethargy and somnolence to stupor and profound coma [2]. Coma may be a presenting feature or develop during the ICU course due to the direct effects of encephalitis, uncontrolled seizures, elevated intracranial pressure (ICP), or as a consequence of sedative medications used to manage agitation or SE. The depth and duration of coma are important prognostic indicators [20]. Respiratory failure is also a common pathway to ICU admission and mechanical ventilation in AE [20]. Respiratory compromise in these patients can arise from several mechanisms, such as central hypoventilation from brainstem issues, aspiration pneumonia due to reduced consciousness or swallowing problems, weak respiratory muscles, or the need for breathing support during seizures or deep sedation [3].

A variety of striking hyperkinetic movement disorders can occur, particularly in NMDAR‐antibody encephalitis, including facial dyskinesias, chorea, and myoclonus [21]. Catatonia, characterized by mutism, stupor, posturing, or stereotypies, can also be a prominent feature [2]. These movement disorders can be severe, persistent, and cause significant distress. Sometimes, they require deep sedation and ICU admission and monitoring for patient safety, prevent complications like exhaustion or rhabdomyolysis, or to manage secondary complications. Recognizing specific movement disorders (e.g., faciobrachial dystonic seizures in LGI1‐antibody encephalitis, orofacial dyskinesias in NMDAR‐antibody encephalitis) can provide vital diagnostic clues in the often‐complex ICU patient, potentially facilitating the rapid initiation of targeted antibody testing and appropriate immunotherapy [4]. Acute agitation, psychosis, and delirium are also common initial or prominent manifestations of AE, particularly in syndromes like NMDAR‐antibody encephalitis [20]. These symptoms significantly complicate ICU management, often necessitating physical restraints or pharmacological sedation, and can mask the emergence of other neurological signs or fluctuations in consciousness [22]. The prominence of psychiatric symptoms can also lead to initial misdiagnosis or delayed neurological consultation, especially if subtle neurological signs are overlooked [23]. In the ICU, distinguishing AE‐driven psychosis or agitation from generic ICU delirium (due to sepsis, metabolic derangements, or medications) is a critical challenge. Atypical, rapidly progressive psychiatric presentations, especially when accompanied by even subtle neurological signs (e.g., new‐onset seizures, mild dysautonomia), should prompt a low threshold for AE investigation.

### 2.4 Diagnostic Strategies for Autoimmune Encephalitis

**FIGURE 3:**
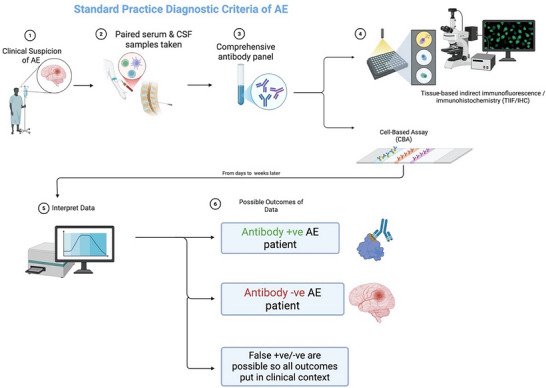
The standard pathway for the diagnosis of AE. Figure 3 was created with BioRender.com. Veenith, T. (2025) https://BioRender.com/zi34cyt.

Diagnosing AE in the ICU presents unique challenges due to the acute nature of the illness, potential communication difficulties with the patient, and the overlap of symptoms with other critical conditions. A systematic and multimodal diagnostic approach is therefore essential (Figure 3). Standardized diagnostic criteria, such as those proposed by Graus and colleagues, offer a framework for classifying patients as having possible, probable, or definite AE [24]. These criteria, applicable even before specific autoantibody results are available, emphasize a subacute onset of symptoms, specific neurological findings, and the reasonable exclusion of alternative diagnoses [4, 24]. The application of these criteria promotes a structured diagnostic process, facilitates early consideration of AE, and enables the initiation of empiric therapy in highly suspicious cases (Figure 3).

AE diagnosis often requires detecting specific neural autoantibodies in both serum and cerebrospinal fluid (CSF). CSF testing is strongly recommended, as CSF analysis can be more sensitive for specific antibodies and generally offers higher specificity [25, 26]. Comprehensive antibody panels are preferred over sequential single‐antibody testing to maximize diagnostic yield and expedite diagnosis [27]. Laboratory methodologies, including cell‐based assays (CBAs) in conjunction with tissue‐based indirect immunofluorescence/immunohistochemistry (TIIF/IHC), if facilities and local resources allow, are considered standard practice to optimize sensitivity and specificity [21]. However, antibody test results can take days to weeks, often necessitating empiric treatment decisions based on clinical suspicion [8]. Clinicians must be aware of the potential for false‐positive or false‐negative results and interpret all antibody findings within the context of the patient's clinical presentation and other paraclinical data. A significant subset of patients may be antibody‐negative, which further complicates the diagnosis [3].

A consolidated overview of key autoantibodies (Table 2) relevant to ICU‐managed AE can be invaluable for clinicians.

**TABLE 2:** Key autoantibodies in ICU‐managed autoimmune encephalitis: targets, syndromes, paraneoplastic links, and diagnostic clues.

Antibody Name (% Antibody Frequency) [28]	Antigen Target & Location	Common Clinical Syndromes in the ICU	Typical Paraneoplastic Association (Cancer Type)	Key Diagnostic Clues in ICU
**Anti‐LGI1** (34%)	Leucine‐rich glioma‐inactivated 1; synaptic, secreted	Limbic encephalitis [13] (memory loss, confusion, psychiatric), faciobrachial dystonic seizures (FBDS)	Infrequent; rarely thymoma, SCLC	FBDS (often pathognomonic); MRI: medial temporal lobe T2/FLAIR hyperintensity (common but can be normal); hyponatremia frequent [1, 18, 22]
**Anti‐NMDAR** (15%)	NR1 subunit of NMDAR; cell surface	RSE, psychosis, agitation, movement disorders (orofacial dyskinesia), dysautonomia, coma, memory loss	Ovarian teratoma [29] (esp. young women), other tumors (rarely)	Often normal MRI initially; EEG: extreme delta brush (specific but not always present), diffuse slowing; CSF pleocytosis common [4]
**Anti‐GAD65** (12%)	Glutamic acid decarboxylase 65; Intracellular enzyme	Stiff‐person syndrome, cerebellar ataxia, limbic encephalitis, epilepsy (often refractory)	Thymoma, SCLC, breast cancer (less common than classic onconeural antibodies)	High antibody titers may correlate with neurological disease; it often requires long‐term immunotherapy [4]
**Anti‐CASPR2** (4%)	Contactin‐associated protein‐like 2; cell surface	Limbic encephalitis, Morvan syndrome (neuromyotonia, dysautonomia, insomnia, cerebellar ataxia)	Thymoma (20%–50%), lung cancer	Peripheral nerve hyperexcitability; MRI: variable, can be normal or show temporal lobe changes [4]
**Anti‐GABA_A_ R**	GABA_A_ receptor subunits; Cell surface	Refractory SE, epilepsy, encephalitis with multifocal cortical/subcortical lesions	Thymoma, other tumors	Severe, often therapy‐refractory seizures; MRI: multifocal T2/FLAIR hyperintensities [1, 18, 22]
**Anti‐GABA_B_ R**	GABA_B_ receptor (B_1_/B_2_ subunits); Cell surface	Limbic encephalitis, prominent early seizures, often SE	Small‐cell lung cancer (SCLC) (∼50%), neuroendocrine tumors	Early and severe seizures; high tumor association mandates urgent screening [4]
**Anti‐AMPA R**	AMPA receptor (GluA1/GluA2 subunits); cell surface	Limbic encephalitis, psychiatric symptoms, seizures	Thymoma, SCLC, breast, ovarian cancer (∼65%–70%)	Relapses are common; memory loss can become irreversible [4]
**Anti‐Hu (ANNA‐1)**	Hu proteins (RNA‐binding proteins); intracellular (onconeural)	Sensory neuronopathy, limbic encephalitis, brainstem encephalitis, cerebellar degeneration	SCLC (>80%), neuroblastoma	T‐cell mediated; poor response to immunotherapy; focus on tumor treatment [8]
**Anti‐Yo (PCA‐1)**	Yo proteins (Purkinje cell cytoplasmic); intracellular (onconeural)	Paraneoplastic cerebellar degeneration	Ovarian, breast cancer (>90%)	T‐cell mediated; poor response to immunotherapy [8]
**Anti‐Ma2 (Ta)**	Ma2 protein; intracellular (onconeural)	Limbic, diencephalic, brainstem encephalitis	Testicular germ cell tumors (young men), lung cancer (older patients)	Often associated with brainstem dysfunction, eye movement abnormalities [30]
**Anti‐DPPX**	Dipeptidyl‐peptidase‐like protein‐6; Cell surface	Prodromal GI symptoms (diarrhea, weight loss), then CNS hyperexcitability, cognitive decline, agitation	Rarely associated with B‐cell lymphomas	Prominent prodromal diarrhea; agitation, tremors, myoclonus; MRI often normal or nonspecific [24, 31]
**Anti‐IgLON5**	IgLON5 cell adhesion molecule; Ccell surface	Sleep disorders (parasomnias, OSA with stridor), bulbar dysfunction, gait instability, movement disorders	No clear tumor association; primary tauopathy found on neuropathology	Chronic, progressive course typical; immunotherapy response often suboptimal; CSF inflammation can occur [32]

Abbreviations: AE: autoimmune encephalitis; AMPA R: α‐amino‐3‐hydroxy‐5‐methyl‐4‐isoxazolepropionic acid receptor; CASPR2: Contactin‐associated protein‐like 2; CSF: cerebrospinal fluid; DPPX: dipeptidyl‐peptidase‐like protein‐6; EEG: electroencephalogram; FBDS: faciobrachial dystonic seizures; GABAA R: γ‐aminobutyric acid type A receptor; GABAB R: γ‐aminobutyric acid type B receptor; GAD65: glutamic acid decarboxylase 65; GI: gastrointestinal; ICU: intensive care unit; IgLON5: immunoglobulin‐like cell adhesion molecule 5; LGI1: leucine‐rich glioma‐inactivated 1; MRI: magnetic resonance imaging; NMDAR: N‐methyl‐d‐aspartate receptor; OSA: obstructive sleep apnea; RSE: refractory status epilepticus; SE: status epilepticus; SCLC: small‐cell lung cancer; antibody frequency percentage has been derived from the article “Autoimmune Encephalitis Criteria in Clinical Practice” by Orozco et al. (2023), a review that includes results from a study of 538 patients with confirmed antibody positive AE.28 The frequency of the four most prevalent subtypes as described by this paper have been included in the table.

Neuroimaging plays a vital role in the diagnostic evaluation of AE in the ICU. Brain magnetic resonance imaging (MRI) is a cornerstone, although it may be normal in a substantial proportion of cases, particularly early in the disease course [24]. When abnormal, characteristic findings include T2‐weighted or FLAIR hyperintensities, often in the medial temporal lobes, or in multifocal areas suggestive of inflammation or demyelination [1, 3], and subtle T2 hyperintensities in the hypothalamus have been reported in IgLON5‐associated disease [33]. A follow‐up MRI should be considered if the initial imaging is negative but clinical suspicion remains high, as abnormalities may evolve [27]. MRI is also crucial for excluding alternative diagnoses. Fluorodeoxyglucose positron emission tomography (FDG‐PET) is emerging as a potentially more sensitive imaging modality in some cases, revealing abnormalities even when MRI is normal or nonspecific [27]. Specific metabolic patterns have been described, and FDG‐PET may serve as a biomarker for early diagnosis and monitoring disease activity [4, 34]. However, the specificity of PET findings is still under investigation, and logistical challenges exist in performing PET scans in unstable ICU patients [11].

Electroencephalography (EEG) is also crucial in the ICU setting for evaluating and managing AE patients. Continuous EEG (cEEG) monitoring is essential for detecting seizures, including nonconvulsive seizures and nonconvulsive status epilepticus (NCSE), and for guiding treatment [35]. Common EEG findings include generalized or focal slowing of background activity, and specific patterns, such as the “extreme delta brush” in NMDAR‐antibody encephalitis, can be highly suggestive [19, 24, 36]. CSF analysis is indispensable for supporting a diagnosis of AE and, critically, for excluding infectious etiologies. Findings suggestive of CNS inflammation include CSF pleocytosis, elevated protein concentration, the presence of CSF‐specific oligoclonal bands, or an elevated IgG index [28]. However, a normal CSF profile does not exclude AE, and CSF should be routinely tested to exclude common infectious etiologies [37].

Diagnosing AE in the ICU is challenging due to the existence of antibody‐negative cases, atypical presentations, and the necessity of excluding a broad range of mimicking conditions. Further complicating the process are potential limitations in the accessibility, cost, and timeliness of comprehensive testing and advanced neuroimaging [38, 39, 40, 41, 42]. A significant proportion of patients who otherwise fit the clinical and paraclinical picture of AE do not have detectable neural autoantibodies with current commercial assays [3]. This “antibody‐negative AE” group likely represents a heterogeneous collection of unidentified antibody‐mediated diseases or other novel autoimmune mechanisms. The paramount importance of multimodal testing, which integrates the clinical phenotype with findings from serology, CSF analysis, EEG, and neuroimaging, cannot be overstated in the ICU management of suspected AE.

### 2.5 Immunotherapeutic Interventions for Autoimmune Encephalitis

**FIGURE 4:**
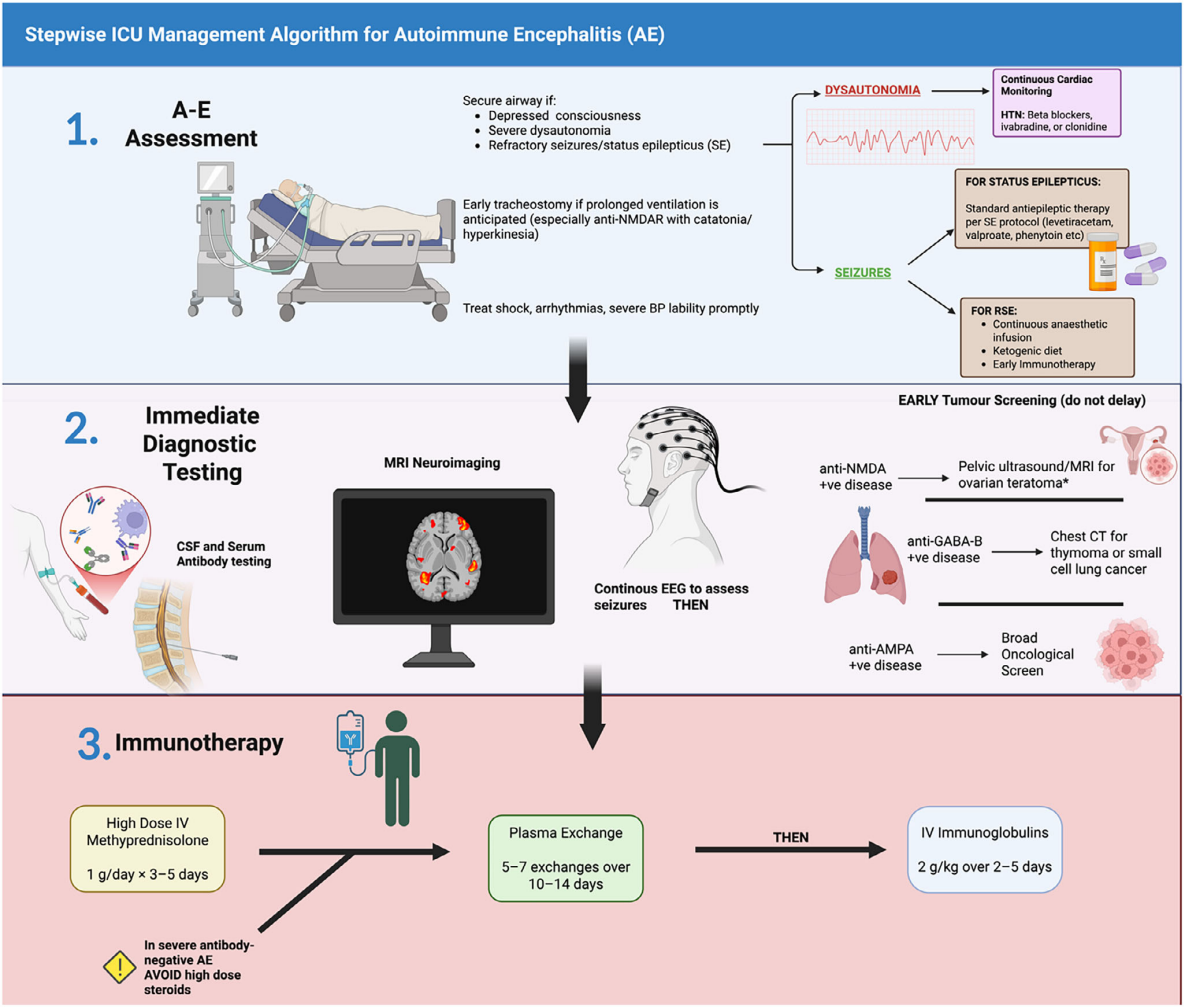
Stepwise management of AE in ICU. *Although testicular teratomas are much less common in males, it is also recognized that ultrasound/MRI imaging is required for the screening of all male AE patients. Figure 4 was created in BioRender. Veenith, T. (2025) https://BioRender.com/60wwnht.

The mainstay of treatment for AE is immunotherapy, which aims to suppress the aberrant immune response and deplete pathogenic autoantibodies [43]. In the ICU setting, where patients are often severely ill, the timely and appropriate administration of these therapies is critical. A principle in managing severe AE is the early initiation of immunotherapy. Numerous observational studies and consensus guidelines emphasize that prompt treatment is associated with improved neurological outcomes, reduced disability, and fewer relapses [25]. Treatment should ideally not be delayed pending definitive autoantibody results. If there is a high index of clinical suspicion for AE and other diagnoses, especially infections, have been reasonably excluded, treatment should begin promptly (Figure 4). Therapy should ideally not be delayed while awaiting definitive autoantibody results [27]. A multidisciplinary team approach, involving neurologists, intensivists, immunologists/rheumatologists, microbiologists, and oncologists (if a paraneoplastic syndrome is suspected), is recommended to guide the complex management of these patients. In many encephalitis patients, differentiating between infectious and autoimmune etiologies may be difficult before CSF analysis; therefore, starting empiric antimicrobials with CNS coverage is always recommended until the infection is excluded. The common practice is to begin CNS doses of intravenous acyclovir and standard coverage for bacterial meningitis, as well as addition of intravenous amoxicillin for vulnerable populations—including elderly, neonatal, or immunocompromised patients to cover for listeria. Antibiotics and acyclovir can later be discontinued if CSF bacterial and HSV/VZV studies return negative.

First‐line immunotherapies typically include high‐dose intravenous corticosteroids, plasma exchange (PLEX), and intravenous immunoglobulin (IVIg). These can be used as monotherapy, sequentially, or increasingly, in combination, particularly in severe cases [25]. Intravenous methylprednisolone (e.g., 1 g daily for 3–5 days, followed by an oral taper) is a common first‐line choice [11]. Corticosteroids exert broad anti‐inflammatory and immunosuppressive effects, including reducing leukocyte trafficking and inflammatory cytokine production, and they have good CNS penetration [44]. While widely used, their efficacy can vary, and some studies suggest that combining PLEX with corticosteroids may yield better outcomes than corticosteroids alone in specific AE populations [25]. However, a critical finding in severe antibody‐negative AE patients in the ICU is that high‐dose corticosteroid therapy was independently associated with poor functional outcomes [25]. ICU‐specific complications of high‐dose steroids are numerous and include hyperglycemia, increased risk of infections, delirium or psychosis, electrolyte disturbances, fluid retention, gastrointestinal bleeding, and steroid‐induced myopathy [25]. Prolonged use necessitates prophylaxis against Pneumocystis jirovecii pneumonia (PJP) [45]. IVIg, typically administered at a total dose of 2 g/kg, divided over 2–5 days, is thought to work through multiple immunomodulatory mechanisms, including neutralization of autoantibodies, modulation of Fc receptor function, inhibition of complement activation, and alteration of B‐cell and T‐cell function [27, 46]. Clinical benefit is often seen within days to weeks [47, 48]. There is randomized controlled trial evidence supporting IVIg efficacy in LGI1/CASPR2 antibody‐positive AE [27]. In the ICU, potential complications include headache, aseptic meningitis, a low risk of thromboembolic events, acute renal failure, fluid overload, and, rarely, anaphylaxis in individuals with IgA deficiency [26, 49]. PLEX involves removing the patient's plasma and replacing it with albumin or fresh frozen plasma, thereby physically removing pathogenic autoantibodies, immune complexes, and other inflammatory mediators from circulation [50]. A typical course consists of 5–7 exchanges performed every other day over 10–14 days [26, 50]. PLEX may be preferred in most patients with particularly severe manifestations, such as rapidly progressive disease, severe dysautonomia, RSE, or central hypoventilation [45]. Clinical improvement can be observed within days to weeks [50]. ICU‐specific complications include hypotension, electrolyte imbalances, coagulopathy, and risks associated with central venous catheter placement [46]. For patients with severe AE, Canadian consensus guidelines recommend initiating combination therapy with high‐dose corticosteroids AND either IVIg or PLEX to maximize the chances of a rapid and robust response in critically ill patients [51] (Table 3).

**TABLE 3:** First‐line immunotherapies for severe autoimmune encephalitis in the ICU: regimens, efficacy summary, and key complications.

Therapy	Typical ICU Regimen	Proposed Mechanism of Action	Summary of Efficacy in Severe AE	Major ICU‐Relevant Complications and Management Pointers
**IV methylprednisolone**	1 g daily IV for 3–5 days, followed by oral prednisone taper (e.g., 1 mg/kg/day)	Broad anti‐inflammatory and immunosuppressive effects; reduces cytokine production, modulates immune cell function, good CNS penetration [44]	Widely used, often initial therapy. May be less effective as monotherapy in severe cases vs. combination. Caution in antibody‐negative AE where it was linked to poor outcomes [3, 25]	Hyperglycemia (monitor glucose, insulin PRN), infections (surveillance, PJP prophylaxis if prolonged), psychosis/delirium, hypertension, electrolyte imbalance, fluid retention, peptic ulcer (prophylaxis), myopathy [25, 45]
**Intravenous immunoglobulin (IVIg)**	Total dose 2 g/kg, given as 0.4 g/kg/day for 5 days or divided over 2–5 days	Neutralizes autoantibodies, Fc receptor modulation, anti‐complementary, B/T cell modulation [27, 46]	Benefit in days to weeks. RCT evidence in LGI1/CASPR2 AE [52]. Often used with steroids.	Headache, aseptic meningitis, thromboembolic events (assess risk, hydration), acute renal failure (avoid sucrose preps), fluid overload (monitor volume status), transfusion reactions, IgA deficiency anaphylaxis (rare) [49]
**Plasma exchange (PLEX)**	5–7 exchanges (1–1.5 plasma volumes per exchange) every other day over 10–14 days	Removal of autoantibodies, immune complexes, inflammatory mediators [44]	Benefit in days to weeks. May be preferred in severe presentations (RSE, dysautonomia). Often combined with steroids.	Hypotension, hypocalcemia (citrate), coagulopathy, electrolyte imbalance, central line complications (infection, thrombosis, pneumothorax, bleeding) [53]

Approximately 20%–50% of AE patients do not respond adequately to first‐line treatments and are considered refractory [54, 55]. For these patients, particularly those in the ICU, escalation to second‐line therapies is necessary. This is typically considered if there is no clinical improvement or if the patient worsens 5–10 days after initiating first‐line treatment in severe AE (a shorter timeframe than the 2–4 weeks often quoted for milder disease, reflecting the urgency in critical care) [56, 57]. The timing of escalation is a crucial decision point; prolonged, ineffective first‐line therapy in the ICU may be detrimental, and an evolving understanding supports earlier consideration of second‐line agents in non‐responders [25]. Rituximab, an anti‐CD20 monoclonal antibody, depletes CD20‐expressing B‐cells and is a preferred second‐line therapy for AE associated with cell‐surface antibodies and for antibody‐negative AE, often due to a relatively favorable efficacy and safety profile compared to cyclophosphamide in these contexts [27, 50, 56]. It has been shown to improve outcomes in refractory cases and can also be used as a maintenance therapy to prevent relapses [50, 58]. Typical adult dosing is 1000 mg IV, repeated in 2 weeks, or 375 mg/m^2^ weekly for 4 weeks. ICU considerations include the risk of infusion reactions, delayed neutropenia, hypogammaglobulinemia, and an increased risk of infections, including reactivation of hepatitis B or C, or tuberculosis (screening is essential before administration). Therapeutic effects typically take weeks to manifest [58]. Seery et al. have suggested that a single course of rituximab not only reduces chance of relapse but prolongs time of the first relapse of disease [59], creating an interest for future research to investigate its use as an adjuvant first‐line therapy alongside corticosteroids and IVIg in management of NMDAR‐antibody encephalitis. Cyclophosphamide, an alkylating agent, exerts broader immunosuppressive effects by targeting both B‐cells and T‐cells [60]. It is preferentially recommended as a second‐line agent for paraneoplastic AE associated with intracellular antibodies, where T‐cell‐mediated damage is prominent [27]. It is also used for other refractory AE cases [25, 54]. Dosing is often pulsed IV (e.g., 600–1000 mg/m^2^ monthly for 3–6 months) [27]. ICU considerations include significant risks of myelosuppression, hemorrhagic cystitis, nausea/vomiting, alopecia, and long‐term risks of infertility and secondary malignancies. Similar to rituximab, its therapeutic effects are not immediate (Summarised in Table 4).

**TABLE 4:** Second‐line and novel immunotherapies for refractory autoimmune encephalitis in the ICU: targets, evidence base, and ICU considerations.

Therapy	Target/Mechanism	Evidence in Refractory AE	Typical Dosing Approach (Adults)	Major ICU‐Relevant Adverse Events and Monitoring
**Rituximab**	CD20 on B‐cells; B‐cell depletion	Preferred second line for cell‐surface and Ab‐negative AE [27]. Improves outcomes. [54, 58]	1000 mg IV x2 (2 weeks apart) OR 375 mg/m^2^ IV weekly ×4	Infusion reactions, neutropenia, hypogammaglobulinemia, infections (HBV/HCV/TB screen prior) [4]
**Cyclo‐phosphamide**	Alkylating agent; B & T cell cytotoxicity	Paraneoplastic AE (intracellular Ab) [27]; other refractory AE [54]	Pulsed IV: 600–1000 mg/m^2^ monthly ×3–6 [21]	Myelosuppression (infection risk), hemorrhagic cystitis (mesna, hydration), N/V, infertility [4]
**Tocilizumab/ Satralizumab**	IL‐6 receptor; blocks IL‐6 signaling	Emerging for refractory AE (esp. NMDARe). Satralizumab in CIELO trial (NCT05503264) [56]	Tocilizumab: e.g., 8 mg/kg IV q4wks. Satralizumab: SC loading then q4wks	Infections, neutropenia, LFT elevation, GI perforation (rare), demyelination (rare) [19]
**Bortezomib**	26S Proteasome; plasma cell depletion	Case reports/series in refractory NMDARe, CASPR2. Generate‐Boost trial (NCT03993262) [53, 58]	SC/IV, e.g., 1.3 mg/m^2^ on days 1, 4, 8, 11 of 21‐day cycle (with dexamethasone) [58]	GI upset, herpes zoster (prophylaxis), hematologic toxicity, neuropathy, cardiac dysfunction [53]
**Inebilizumab**	CD19 on B‐cells, plasmablasts, some plasma cells; broader B‐cell depletion	ExTINGUISH trial (NCT04372615) for moderate‐severe NMDARe [58]	300 mg IV on Day 1 and Day 15 (trial protocol) [62]	Infusion reactions, infections (similar to anti‐CD20)
**Rozanolixizumab**	Neonatal Fc receptor (FcRn); promotes IgG degradation	*LEGIONE trial (NCT04875975) for LGI1‐AE [58]	Subcutaneous, weight‐based dosing (trial protocol)	Headache, infections, and potential rapid IgG reduction

Abbreviations: Ab: antibody; AE: autoimmune encephalitis; CASPR2: Contactin‐associated protein‐like 2; GI: gastrointestinal; HBV: hepatitis B virus; HCV: hepatitis C virus; ICU: intensive care unit; IL‐6: interleukin‐6; IV: intravenous; LFT: liver function tests; LGI1: leucine‐rich glioma‐inactivated 1; NMDARe: N‐methyl‐d‐aspartate receptor encephalitis; N/V: nausea/vomiting; SC: subcutaneous; TB: tuberculosis.*it is critical to note that the LEGIONE trial has since terminated at the Phase 2 stage due to significant challenges in patient recruitment.

IL‐6 pathway inhibitors, such as tocilizumab and satralizumab, offer a targeted immunomodulatory approach by blocking interleukin‐6 (IL‐6), a key cytokine involved in B‐cell differentiation, T‐cell proliferation, and plasma cell survival. There is emerging evidence for their efficacy in AE refractory to other treatments, including NMDAR‐antibody encephalitis [25]. Satralizumab is currently being investigated in the CIELO phase 3 clinical trial (NCT05503264) for both NMDAR and LGI1 antibody‐associated AE [61]. ICU considerations include risks of infection, neutropenia, elevated liver enzymes, and, rarely, gastrointestinal perforation or demyelinating disorders [25]. Proteasome inhibitors, such as bortezomib, target and deplete antibody‐producing plasma cells, which are often resistant to B‐cell depleting therapies like rituximab [60]. Bortezomib has shown promise in case reports and small series of AE patients refractory to first‐ and second‐line therapies, particularly in those with NMDAR‐antibody encephalitis and CASPR2‐antibody associated AE [61]. The Generate‐Boost phase 2 clinical trial (NCT03993262) is evaluating bortezomib in seropositive AE patients who have previously received rituximab [61]. ICU considerations include gastrointestinal side effects, risk of herpes zoster reactivation, hematological toxicity, peripheral neuropathy, and cardiovascular dysfunction [56, 61]. Other B‐cell/plasma cell‐targeted therapies, like inebilizumab (anti‐CD19 monoclonal antibody), and neonatal Fc receptor (FcRn) inhibitors, such as rozanolixizumab, are also being investigated [61]. The increasing array of targeted immunotherapies entering clinical trials signals a shift toward more personalized and potentially less broadly immunosuppressive approaches for AE. In the ICU, this could translate to more effective rescue options for refractory patients, possibly with improved safety profiles compared to conventional broad immunosuppressants. However, these novel agents will also require careful patient selection, often guided by biomarkers, and vigilant management of their unique toxicity profiles.

The aggressive immunotherapies required for severe AE in the ICU create a “double‐edged sword” scenario: these treatments can be lifesaving but carry substantial risks of severe complications in already physiologically vulnerable patients. General principles for managing these adverse events include prophylaxis for opportunistic infections (e.g., PJP with trimethoprim‐sulfamethoxazole), screening for latent infections (e.g., viral hepatitis, HIV, tuberculosis) prior to initiating potent immunosuppression, vigilant monitoring for specific complications related to each agent (e.g., blood counts, liver and renal function, electrolytes, glucose levels, and signs of infection), and a careful and ongoing risk‐benefit assessment, particularly in patients with multiple comorbidities, active infections, or those who are profoundly immunosuppressed (Figure 4).

### 2.6 General ICU Management Considerations

Beyond immunotherapy, ICU management of AE patients involves aggressive treatment of specific neurological emergencies and comprehensive supportive care. RSE in AE demands a multifaceted approach. Initial management involves emergent administration of benzodiazepines, and if seizures persist, second‐line ASMs are added, such as intravenous valproate, levetiracetam, fosphenytoin/phenytoin, or phenobarbital [63]. For seizures refractory to these, continuous intravenous infusions of anesthetic agents like midazolam, propofol, pentobarbital/thiopental, or ketamine are employed, guided by continuous EEG monitoring [4]. Ketamine, an NMDAR antagonist, is increasingly used in RSE, which may be particularly relevant in NMDAR‐antibody encephalitis [60]. To effectively control seizures in AE‐associated RSE, aggressive immunotherapy must be given concurrently with ASMs, emphasizing that addressing the autoimmune cause is paramount. In cases of super‐RSE, other interventions may be considered, including ketogenic diet, therapeutic hypothermia, electroconvulsive therapy (ECT), CSF drainage, and, rarely, emergency resective epilepsy surgery if a focal onset is identified [64, 65]. Management of NORSE or Febrile Infection‐Related Epilepsy Syndrome (FIRES), which can be manifestations of AE, often requires a specialized multidisciplinary team and early, aggressive immunotherapy [62].

Severe dysautonomia is a common and serious complication in ICU‐managed AE patients, potentially reflecting direct immune attack on autonomic control centers or widespread inflammation [66]. It can manifest as labile blood pressure, cardiac arrhythmias, temperature instability, central respiratory disturbances, gastrointestinal dysmotility, sialorrhea, and hyperhidrosis [66, 67]. Management is primarily supportive and symptomatic. Continuous hemodynamic monitoring is essential. Severe bradycardia or asystole may necessitate temporary or permanent cardiac pacing [20, 67, 68]. Hypotension may require fluid resuscitation and vasopressor support, while hypertensive crises are managed with intravenous antihypertensive agents [17, 64]. Tachyarrhythmias may be treated with beta‐blockers or other antiarrhythmic drugs, with caution regarding potential proarrhythmic effects or exacerbation of blood pressure lability. Hyperthermia should be managed aggressively with external or internal cooling methods; intravenous dantrolene has also been reported to be used [17, 64]. Careful attention to fluid balance and correction of electrolyte abnormalities is crucial. Medications known to exacerbate autonomic dysfunction should be avoided or used with extreme caution. The presence of severe dysautonomia can be a marker of disease severity and a direct contributor to mortality and morbidity, potentially influencing decisions about escalating immunotherapy [20, 62].

Mechanical ventilation is frequently required in severe AE due to depressed level of consciousness, central hypoventilation, failure to protect the airway during SE or severe agitation, or respiratory muscle weakness. Weaning from mechanical ventilation can be particularly challenging and prolonged in AE patients due to persistent encephalopathy, the development of critical illness neuromyopathy, ongoing central respiratory drive issues, or recurrent seizures [69]. Standard ICU weaning protocols are applicable, including daily assessment of weaning readiness, spontaneous breathing trials, and a multidisciplinary team approach involving an Intensivist, Neurologist, respiratory therapists/physiotherapists, and nurses. The focus is on reversing all reversible causes of ventilator dependence, optimizing respiratory mechanics, and gradually reducing ventilatory support. Early consideration of tracheostomy may be warranted in patients anticipated to require prolonged mechanical ventilation to facilitate weaning, improve comfort, and minimize sedation needs [15, 20, 62].

Severe agitation, delirium, and psychosis are common and distressing symptoms in AE, particularly in NMDAR‐antibody encephalitis, and can significantly complicate ICU care [70]. Management is challenging as patients with AE may exhibit heightened sensitivity or paradoxical reactions to conventional antipsychotics, with an increased risk of developing neuroleptic malignant syndrome (NMS)‐like symptoms or severe extrapyramidal side effects [66]. If pharmacological intervention is necessary, atypical antipsychotics (e.g., olanzapine, quetiapine, risperidone) are generally preferred, initiated at low doses and titrated cautiously [66]. Benzodiazepines (e.g., lorazepam) can be helpful in acute agitation and anxiety, and clonidine may help with agitation and sleep disturbances [66]. It is crucial to remember that immunotherapy targeting the underlying AE is the definitive treatment for these neuropsychiatric manifestations. Nonpharmacological management, including environmental modifications, frequent reorientation, early mobilization, ensuring patient safety, and involving family members, are also shown as critical adjunctive measures [71] In the ICU setting, it is essential to diligently rule out other common causes of delirium and agitation, such as sepsis, metabolic derangements, hypoxia, drug withdrawal, or side effects of different medications, before attributing these symptoms solely to AE. The complexity arises because AE itself can present with features mimicking NMS (hyperthermia, rigidity, autonomic dysregulation, altered consciousness) [66], making the distinction from drug‐induced NMS difficult.

Comprehensive supportive care is crucial. Early initiation of enteral nutrition is generally preferred to maintain gut integrity and provide adequate caloric support. Gastroparesis can occur in AE [72], potentially requiring prokinetic agents or post‐pyloric feeding. AE patients are at high risk of nosocomial infections due to immunosuppressive therapies, invasive devices, and prolonged immobility. Strict adherence to infection prevention bundles and vigilant surveillance for infections are critical. Standard ICU protocols for VTE prophylaxis should be implemented, with careful consideration of bleeding risks, especially in patients undergoing PLEX or those with coagulopathy. Regular repositioning and use of pressure‐relieving surfaces are essential for preventing pressure ulcers in immobilized patients. If elevated intracranial pressure (ICP) is present or suspected, standard management strategies should be employed, guided by clinical assessment and, if available, ICP monitoring.

### 2.7 Syndrome‐Specific Considerations in ICU Management

While general principles of neurocritical care and immunotherapy apply to all severe AE patients, specific autoantibody syndromes often have unique clinical features, tumor associations, and treatment responses that warrant tailored ICU management strategies.

Patients with NMDAR‐antibody encephalitis frequently experience prolonged ICU course characterized by high rates of mechanical ventilation, RSE, severe dysautonomia, and prominent hyperkinetic movement disorders or catatonia. Elevated ICP can also complicate management [73]. A significant proportion of young women with this condition have an underlying ovarian teratoma, necessitating early detection and surgical resection, which is critical for better neurological outcomes and reduced relapse rates [8]. Tumor screening should be initiated promptly upon suspicion or confirmation of NMDAR‐antibody encephalitis. In these critically ill patients, indicators of increased mortality include older age, prolonged symptom duration before treatment, the presence of medical comorbidities, and the necessity of a tracheostomy [73]. Conversely, lower CSF inflammatory markers and early immunotherapy initiation are independent predictors of good neurological outcome [74]. Many patients respond well to immunotherapy, but recovery is often slow and protracted [8]. The ketogenic diet has been reported as a beneficial adjunctive therapy for RSE in this population [69].

LGI1‐antibody encephalitis may require ICU admission for management of SE, severe hyponatremia, or profound cognitive and behavioral disturbances [12, 52]. Faciobrachial dystonic seizures (FBDS) are highly characteristic and often precede or accompany limbic encephalitis, serving as a key diagnostic clue. Hyponatremia is common and can complicate ICU management [75]. Patients generally respond well to first‐line immunotherapies and ASMs for seizure control. There is RCT evidence supporting the efficacy of IVIg in LGI1/CASPR2‐antibody positive patients [27]. Some reports suggest that AE associated with pathogenic IgG4 subclass antibodies may respond less robustly to IVIg [24]. Relapses can occur, and factors associated with poorer long‐term outcomes include poor initial treatment response, disease relapse, advanced age at onset, and CSF abnormalities [52]. Persistent cognitive impairment is common even with good seizure control [49].

Patients with CASPR2‐antibody encephalitis may require ICU care for severe manifestations of limbic encephalitis, Morvan syndrome, or refractory seizures [21, 76]. Pain and other symptoms of peripheral nerve hyperexcitability are also prominent, at a greater extent than in comparison with LGI1‐antibody encephalitis [77, 78]. Thymoma is found in a small proportion of these patients, and myasthenia gravis can co‐occur [73, 78]. A favorable response to immunotherapy is generally observed, and second‐line agents may be used for refractory cases [18, 72, 73]. Many patients achieve good outcomes, but an underlying malignancy may indicate a poorer prognosis [73]. GABA_A_R‐antibody encephalitis is characteristically associated with therapy‐refractory seizures and SE, often requiring aggressive ICU management [79]. MRI often reveals multifocal T2/FLAIR hyperintensities [74]. Associated triggers include tumors (mainly thymomas) and antecedent HSVE. Treatment strategies align with those for other cell‐surface antibody‐mediated AEs. A monophasic course has been reported, but achieving complete seizure freedom can be challenging [74]. Eighty percent of patients with GABA_A_R‐antibody encephalitis demonstrate partial or complete recovery [80].

Patients with GABA_B_R‐antibody encephalitis often present with early‐onset seizures, frequently progressing to RSE, necessitating ICU admission. Symptoms of limbic encephalitis are also common [81]. There is a very high association with small‐cell lung cancer and other neuroendocrine tumors, mandating urgent and comprehensive tumor screening [82, 83]. Higher mortality rates have been reported, and older age at onset and the presence of an underlying tumor are independent predictors of poorer long‐term outcomes. Conversely, maintenance immunotherapy for at least 6 months has been associated with more favorable outcomes. [77]. AMPA receptor antibody encephalitis manifests as limbic encephalitis, seizures, and prominent psychiatric features [78]. ICU admission may be required for SE or severe encephalopathy. A high frequency of underlying cancer is reported, and treatment with immunotherapy and appropriate oncologic therapy can lead to improvement [83, 84]. However, relapse is common, and memory loss can become persistent and irreversible, particularly after multiple relapses [78, 79]. In the broader context of ICU‐managed AE, shorter symptom duration before immunotherapy initiation may predict a better outcome, while the development of sepsis is associated with a worse outcome [85].

Rarer syndromes also have specific ICU implications. IgLON5 encephalitis typically presents as a chronic, progressive disorder with a unique constellation of symptoms including a complex and unique sleep disorder, bulbar dysfunction, gait instability, and movement disorders [32]. Acute encephalitic episodes can occur, potentially requiring ICU admission for severe neurological deterioration or respiratory failure [29]. The response to immunotherapy is generally suboptimal, and neuropathological studies reveal a distinctive neuronal tauopathy [32, 33]. DPPX encephalitis often begins with prominent prodromal gastrointestinal symptoms, followed by CNS hyperexcitability, cognitive dysfunction, and sleep disorders [76]. ICU admission may be necessary for managing severe agitation or refractory seizures. Association with underlying cancer is rare [86, 87]. Patients often respond to immunotherapy, but recovery can be slow, and relapses can occur [86, 87, 88]. GAD65‐associated neurological syndromes include stiff‐person syndrome, cerebellar ataxia, limbic encephalitis, and epilepsy that can be difficult to treat [89]. Seizures can be refractory, potentially requiring ICU care, and the response to immunotherapy can be variable [28, 89].

The key neurological signs associated with these syndromes can predict specific ICU challenges and resource requirements, such as the need for prolonged EEG monitoring, particular choices of ASMs, or early consideration of tracheostomy for airway compromise. Due to the varied effectiveness of immunotherapy and different risks of relapse among these syndromes, planning for ICU discharge and discussing long‐term prognosis requires an individualized approach. This means that strategies for maintenance immunomodulation must be explicitly tailored to the identified autoantibody.

### 2.8 Prognostication and Outcomes of ICU‐Treated Autoimmune Encephalitis

Prognosis and outcomes for patients with AE requiring ICU admission are complex and variable, presenting a significant challenge for clinicians. Predicting the trajectory of recovery and long‐term functional status is crucial for guiding therapeutic interventions, managing patient and family expectations, and planning post‐ICU care. A wide array of factors influences both short‐term outcomes, such as ICU mortality and functional status at hospital discharge, and long‐term outcomes, including neurological and cognitive recovery, as well as overall survival.

Several negative prognostic indicators have been consistently identified in AE patients treated in the ICU. Delays in initiating immunotherapy are strongly associated with poorer outcomes [90], as is a lack of response to initial first‐line treatments [91]. Interestingly, in severe antibody‐negative AE requiring ICU care, treatment with high‐dose corticosteroids was paradoxically linked to poor functional outcomes [3]. Disease severity markers such as prolonged ICU length of stay, the need for mechanical ventilation, the presence of SE, severe disturbances in consciousness, or significant autonomic dysregulation all predict worse outcomes [4]. Higher scores on disability scales like the modified Rankin Scale (mRS) or the Clinical Assessment Scale in Autoimmune Encephalitis (CASE) at the onset of illness or ICU admission also correlate with a less favorable prognosis [14]. Comorbidities, including underlying malignancy (particularly in paraneoplastic syndromes), severe sepsis, cerebral oedema, thrombocytopenia [92], and anemia on hospital admission [16], are also negative factors. Patient age at onset is relevant, with older patients often experiencing poorer outcomes across various AE subtypes [3]. The specific type of antibody is also a significant determinant; antibodies against intracellular antigens generally predict a worse prognosis and reduced response to immunotherapy compared to those targeting cell‐surface antigens. In the specific context of antibody‐negative AE in the ICU, acute respiratory failure at admission and the presence of dyskinesia/dystonia have been identified as independent predictors of poor outcome [3]. Conversely, positive prognostic factors include the early and aggressive initiation of immunotherapy, which is strongly linked to better neurological outcomes, especially in conditions such as NMDAR‐antibody encephalitis when initiated within 8 days of ICU admission [74]. A favorable clinical response to first‐line immunotherapy is another strong predictor of good long‐term prognosis [91]. In NMDAR‐antibody encephalitis, lower levels of inflammation in the CSF, indicated by a lower white blood cell count, have been associated with better outcomes [70]. The consistent finding that early immunotherapy improves outcomes underscores the critical concept that “time is brain” in neuroinflammatory conditions, emphasizing the need for rapid diagnosis and treatment initiation in the ICU to mitigate irreversible neuronal damage.

To aid in assessing severity and predicting outcomes, several scoring systems are utilized. The mRS is a widely used measure of global disability, with scores above 2 or 3 often defining a poor outcome in studies [3]. CASE is a more specific tool that evaluates severity across nine neurological domains, providing a more detailed assessment that correlates well with the mRS and helps track changes over time [14, 93]. For NMDAR‐antibody encephalitis, the NEOS score can predict functional status at one year by incorporating factors like ICU admission, treatment delay, lack of early clinical improvement, abnormal MRI, and CSF white blood cell count [94, 95]. Other scores, such as the Antibody Prevalence in Epilepsy (APE) and Response to Immunotherapy in Epilepsy (RITE) scores, may help identify an immune etiology and predict treatment response in AE presenting primarily with seizures [89, 90].

Mortality rates among AE patients admitted to the ICU are substantial, although they vary depending on the study cohort and specific AE subtype, ranging from approximately 12% to 40% [4]. For instance, a study focusing on severe antibody‐negative AE in the ICU reported a 6‐month mortality rate of 26.2% [3]. An earlier study on all‐cause encephalitis in the ICU found a mortality rate of 18.45%, with cerebral oedema, SE, and thrombocytopenia being predictors of death [92]. The primary causes of death in this critically ill population often include severe infections (exacerbated by immunosuppression and prolonged ICU stays), multiple organ dysfunction syndrome, RSE, and complications arising from protracted immobility or severe autonomic dysfunction [4].

Survival after severe AE requiring ICU care frequently leads to a protracted recovery marked by significant long‐term neurological, cognitive, and psychiatric sequelae [96]. It is essential to recognize that while some survivors may achieve a “good functional outcome” based on broad scales like the mRS (score ≤2 or ≤3), they can still experience debilitating cognitive and psychiatric impairments that significantly impact their quality of life. This highlights a limitation of current global functional scales in fully capturing the burden of post‐AE disability. Common long‐term issues include cognitive deficits, such as difficulties with memory, attention, executive functions, and processing speed, which can be persistent despite potential slow improvement over time [25]. Psychiatric sequelae like depression, anxiety, mood lability, fatigue, and sleep disturbances are also frequently reported and can hinder functional recovery [22]. Residual neurological deficits, including ongoing seizures, focal weakness, ataxia, or speech problems, can persist. These combined impairments often lead to difficulties in returning to work or school, managing finances, and participating in social activities, resulting in a diminished overall quality of life [4, 22]. The risk of relapse in AE varies by antibody type, with rates ranging from 10% to 62% in some cohorts [91], necessitating careful management of immunotherapy.

The recovery journey for patients surviving severe AE, results in an ICU stay that extends well beyond hospital discharge, often requiring comprehensive multidisciplinary rehabilitation and long‐term immunomodulatory therapy. Rehabilitation, ideally starting early and continuing for months or years, is crucial for addressing the physical, cognitive, emotional, and social challenges survivors face. A multidisciplinary team comprising physiotherapy, occupational therapy, speech‐language pathology, neuropsychology, psychiatry, psychology, nursing, and social work is essential [97]. Physiotherapy focuses on restoring strength and mobility; occupational therapy helps regain independence in daily activities; speech‐language pathology addresses communication and swallowing issues; neuropsychology provides cognitive assessment and rehabilitation strategies; and psychiatry/psychology manages psychiatric sequelae [92]. Specialized rehabilitation may also be needed for specific deficits. The transition to long‐term care is often fragmented, highlighting the need for dedicated “post‐AE recovery” clinics to coordinate immunosuppression, manage neuropsychiatric issues, and direct rehabilitation [92].

Many patients require long‐term immunosuppression to prevent relapses, particularly those with severe or relapsing disease or those who relapsed during immunotherapy tapering [92]. Common agents include oral corticosteroids, IVIg, rituximab, mycophenolate mofetil, or azathioprine. The optimal duration of maintenance therapy is not definitively established and is individualized, typically lasting at least 1–3 years after stability [92]. Regular monitoring for adverse effects of immunosuppressants is necessary. Antibody titers alone are generally not reliable guides for therapy duration or relapse prediction, with clinical assessment remaining paramount. However, persistently high or rising titers may support continuing or intensifying immunotherapy. The uncertainty in long‐term immunosuppression highlights a need for research into biomarkers that can predict relapse or guide therapy tapering [92].

Addressing persistent cognitive, psychiatric, and sleep disturbances is vital for improving long‐term quality of life [22]. Cognitive deficits are managed with rehabilitation, compensatory strategies, and environmental modifications. Psychiatric symptoms are treated with psychotherapy and/or pharmacotherapy [92]. Sleep disturbances may require behavioral interventions or medication [22]. Patient and family education and support groups are crucial components of long‐term care, helping them navigate the challenging recovery process [92].

### 2.9 Novel Diagnostics, Therapeutics, and Research Priorities for the ICU

The landscape of AE is undergoing rapid transformation driven by dedicated research efforts. The primary focus areas include developing more precise and rapid diagnostic methods, creating highly effective and targeted therapeutic interventions, and deepening understanding of the disease's long‐term effects, particularly in individuals with severe illness requiring ICU admission. This ongoing research aims to improve outcomes for this critically ill patient population.

There is a critical need to identify accessible, rapid diagnostic biomarkers with high sensitivity and specificity for AE, primarily to facilitate early diagnosis and treatment initiation in the acute ICU setting [98]. Promising emerging biomarkers include Neurofilament Light Chain (NfL), which serves as an indicator of neuronal damage and holds potential for assessing disease activity, monitoring treatment response, and predicting neurological outcomes in AE [99]. Advanced autoantibody assays, such as novel ELISAs with enhanced sensitivity, are being developed to detect low‐abundance autoantibodies, such as anti‐NMDAR IgG, in recovering patients [100]. Moreover, exploratory research utilizing “‐omics technologies” (proteomics, metabolomics, and genomics) on CSF and blood samples is underway to uncover novel diagnostic hallmarks, prognostic markers, and insights into AE subtypes [98]. Cytokine and chemokine profiling in CSF or serum, with examples such as elevated CSF IL‐17A in critically ill AE patients or the role of IL‐6, may also serve as biomarkers of disease severity, immune pathway activation, or predictors of response to targeted immunotherapies [14, 25]. The advent of more sensitive antibody detection methods and non‐antibody biomarkers like NfL has the potential to significantly reduce the time to diagnosis for AE in the ICU, potentially decreasing the reliance on empiric treatment, enabling earlier targeted therapy based on confirmed diagnoses or specific pathogenic pathways, and improving prognostication, thereby impacting ICU resource utilization and, crucially, patient outcomes.

A significant focus in current AE research is the development of next‐generation immunotherapies that are more targeted, aiming for improved efficacy with fewer side effects compared to broad immunosuppressants. This represents a potential shift towards precision medicine in AE, offering hope for more effective rescue therapies for refractory cases in ICU patients, potentially with fewer global immunosuppressive side effects. However, this will necessitate sophisticated biomarker‐driven patient selection and careful management of the unique toxicities associated with these novel agents. These emerging therapies include B‐cell targeted therapies beyond rituximab, such as anti‐CD19 monoclonal antibodies like inebilizumab, which target a broader range of B‐cells [61]. Plasma cell targeted therapies, such as proteasome inhibitors like Bortezomib, are being investigated for their ability to deplete long‐lived plasma cells that produce autoantibodies and are often resistant to B‐cell depletion [60, 61]. Cytokine pathway inhibitors, specifically IL‐6 receptor inhibitors such as tocilizumab and satralizumab, are being explored due to the role of IL‐6 in inflammation and autoantibody production [25, 61]. FcRn inhibitors, such as rozanolixizumab, are under investigation for their ability to accelerate the clearance of pathogenic IgG autoantibodies [58]. Innovative approaches like Chimeric Antigen Receptor T‐cell (CAR‐T) therapy, including highly selective Chimeric Autoantibody Receptor T‐cells (CAAR‐T cells) targeting specific autoantibody‐producing B‐cells, are also being explored [61, 101]. In addition, receptor blocking or modulating strategies, such as positive allosteric modulators of the NMDAR or monoclonal antibodies designed to block NMDAR and prevent autoantibody binding, are in development [58]. While not yet in advanced AE‐specific trials, as mentioned, complement inhibitors are also a potential future therapeutic avenue, given the role of complement activation in some CSAab‐mediated AEs.

Several necessary clinical trials are currently underway, specifically addressing severe or refractory AE. The ExTINGUISH trial (NCT04372615) is a phase 2B, randomized, double‐blind, placebo‐controlled study evaluating inebilizumab (anti‐CD19) as an adjunct to first‐line therapies for acute moderate‐to‐severe NMDAR‐antibody encephalitis. The CIELO trial (NCT05503264) is a phase 3, randomized, double‐blind, placebo‐controlled trial investigating satralizumab (anti‐IL‐6R) in patients with NMDAR/LGI1‐antibody encephalitis, encompassing both new‐onset and refractory cases. The Generate‐Boost trial (NCT03993262) is a phase 2, randomized, double‐blind, placebo‐controlled trial assessing bortezomib (proteasome inhibitor) in adults with moderate‐to‐severe seropositive AE who have previously received rituximab. Furthermore, the LEGIONE trial (NCT04875975) is a phase 2 study of rozanolixizumab (FcRn inhibitor) in adults with LGI1‐associated AE.

Addressing the existing knowledge gaps and improving care for critically ill AE patients necessitates a collaborative research effort with defined future priorities. These priorities include the development and validation of rapid, ideally point‐of‐care, diagnostic tests for common and clinically relevant AE‐associated autoantibodies. There is a significant need for well‐designed, prospective, randomized controlled trials comparing different immunotherapy regimens (first‐line, second‐line, and novel agents) specifically in patients with severe AE, including substantial cohorts of ICU‐managed patients, as current trials often focus on broader populations [58]. Identifying and validating early prognostic biomarkers (integrating clinical, imaging, CSF, and blood‐based markers) is crucial for better risk stratification, guiding therapy intensity, and informing prognostic discussions in the ICU. Research into adjunctive neuroprotective strategies aimed at mitigating neuronal injury and promoting recovery during the acute phase of neuroinflammation in the ICU is also a priority. Studies focusing on the optimal management of specific AE‐related neurological emergencies (e.g., RSE, dysautonomia) in the ICU setting are needed to develop evidence‐based protocols. The development and validation of standardized, multidimensional outcome measures sensitive to the cognitive, psychiatric, and quality‐of‐life sequelae relevant to ICU survivors of AE is essential. Intensified research into the pathophysiology, diagnostic markers, and targeted treatments for antibody‐negative AE, a significant challenge in critical care, is vital. Finally, the establishment of international registries and collaborative research networks for severe AE in the ICU is essential to pool data, share expertise, and facilitate the conduct of clinical trials in this relatively rare but high‐impact patient population.

## 3 Conclusion

AE represents a critical and increasingly recognized neurological emergency that frequently culminates in ICU admission due to a spectrum of severe, life‐threatening manifestations. The diagnostic and therapeutic pathways for these patients are complex, often demanding rapid clinical decision‐making under conditions of uncertainty, particularly in the early stages of ICU care. The heterogeneity of AE syndromes, the potential for delayed diagnosis, and the severity of associated neurological complications contribute to significant morbidity and mortality. The management of AE in the ICU is, therefore, a rapidly evolving subspecialty at the dynamic intersection of neurocritical care, neuroimmunology, and general critical care. Its complexity demands not only continued research but also specialized training and the development of dedicated clinical pathways within ICU settings to optimize patient care.

This review underscores that prompt recognition, meticulous exclusion of mimicking conditions, and the early, aggressive initiation of appropriate immunotherapy are paramount for improving outcomes in critically ill AE patients. A highly coordinated, multidisciplinary approach, involving intensivists, neurologists, immunologists/rheumatologists, oncologists, and rehabilitation specialists, is essential throughout the patient's journey, from acute ICU management to long‐term recovery and relapse prevention.

Despite significant advances in recent years, substantial knowledge gaps persist. Continued research is imperative to refine diagnostic algorithms for use in the ICU, establish the comparative effectiveness of various immunotherapeutic strategies through robust clinical trials (especially those including critically ill cohorts), and develop novel, more targeted, and safer therapies. In addition, a greater understanding of the mechanisms driving antibody‐negative AE, which constitutes a major frontier, is crucial for improving care for this challenging subgroup of patients. Enhanced efforts are also needed to identify reliable early prognostic biomarkers and to develop effective interventions for the common and often debilitating long‐term cognitive, psychiatric, and functional sequelae experienced by ICU survivors. The ultimate aim of these endeavors is to reduce mortality, minimize disability, and substantially enhance the quality of life for all patients afflicted by severe AE.

## Conflicts of Interest

The authors declare no conflicts of interest.
